# Genesis of Influenza A(H5N8) Viruses

**DOI:** 10.3201/eid2308.170143

**Published:** 2017-08

**Authors:** Rabeh El-Shesheny, Subrata Barman, Mohammed M. Feeroz, M. Kamrul Hasan, Lisa Jones-Engel, John Franks, Jasmine Turner, Patrick Seiler, David Walker, Kimberly Friedman, Lisa Kercher, Sajeda Begum, Sharmin Akhtar, Ashis Kumar Datta, Scott Krauss, Ghazi Kayali, Pamela McKenzie, Richard J. Webby, Robert G. Webster

**Affiliations:** National Research Centre, Giza, Egypt (R. El-Shesheny);; St. Jude Children’s Research Hospital, Memphis, Tennessee, USA (R. El-Shesheny, S. Barman, J. Franks, J. Turner, P. Seiler, D. Walker, K. Friedman, L. Kercher, S. Krauss, P. McKenzie, R.J. Webby, R.G. Webster);; Jahangirnagar University, Savar, Dhaka, Bangladesh (M.M. Feeroz, M.K. Hasan, S. Begum, S. Akhtar, A.K. Datta);; University of Washington, Seattle, Washington, USA (L. Jones-Engel);; University of Texas Health Sciences Center, Houston, Texas, USA (G. Kayali);; Human Link, Hazmieh, Lebanon (G. Kayali)

**Keywords:** Bangladesh, central Asian flyway, clade 2.3.4.4, highly pathogenic avian influenza A(H5N8), viruses, Egypt, United States, influenza, respiratory infections, zoonoses

## Abstract

Highly pathogenic avian influenza A(H5N8) clade 2.3.4.4 virus emerged in 2016 and spread to Russia, Europe, and Africa. Our analysis of viruses from domestic ducks at Tanguar haor, Bangladesh, showed genetic similarities with other viruses from wild birds in central Asia, suggesting their potential role in the genesis of A(H5N8).

Highly pathogenic avian influenza (HPAI) viruses of the H5 subtype remain a serious concern for poultry and human health. The Gs/GD lineage of HPAI A(H5N1) viruses continues to circulate and spread, and the hemagglutinin (HA) genes have diversified into multiple genetic clades. H5 clade 2.3.4.4 of the H5N8 subtype was first detected in domestic poultry in China in 2010; by 2014, this virus had caused multiple outbreaks among domestic ducks, chickens, geese, and wild birds in South Korea and subsequent outbreaks in Japan, China, Europe, and North America (*1*,*2*). During these outbreaks, 2 distinct clusters of HPAI A(H5N8) viruses were identified: group A viruses were detected in China in early 2014 and later in South Korea, Japan, Taiwan, Canada, the United States, and Europe; group B viruses were detected only in China in 2013 and South Korea in 2014 (*3*,*4*). Co-circulation of group A viruses with low pathogenicity avian influenza (LPAI) viruses led to new reassortants, including H5N1, H5N2, and H5N8 (*3*).

In late May 2016, a novel reassortant group B HPAI A(H5N8) clade 2.3.4.4 virus was detected in a wild bird in UVs-Nuur Lake in the Republic of Tyva, Siberia (*5*). As of March 2017, the virus had spread across most European countries, the Middle East, and Africa (*6*). To better understand the evolution and origin of the novel HPAI A(H5N8) viruses, we sequenced and analyzed the full genomes of LPAI viruses isolated from wild and free-ranging domestic ducks in the Tanguar haor area of Bangladesh, located in the central Asian flyway, and compared them with the novel HPAI A(H5N8) viruses.

## The Study

**Figure 1:**
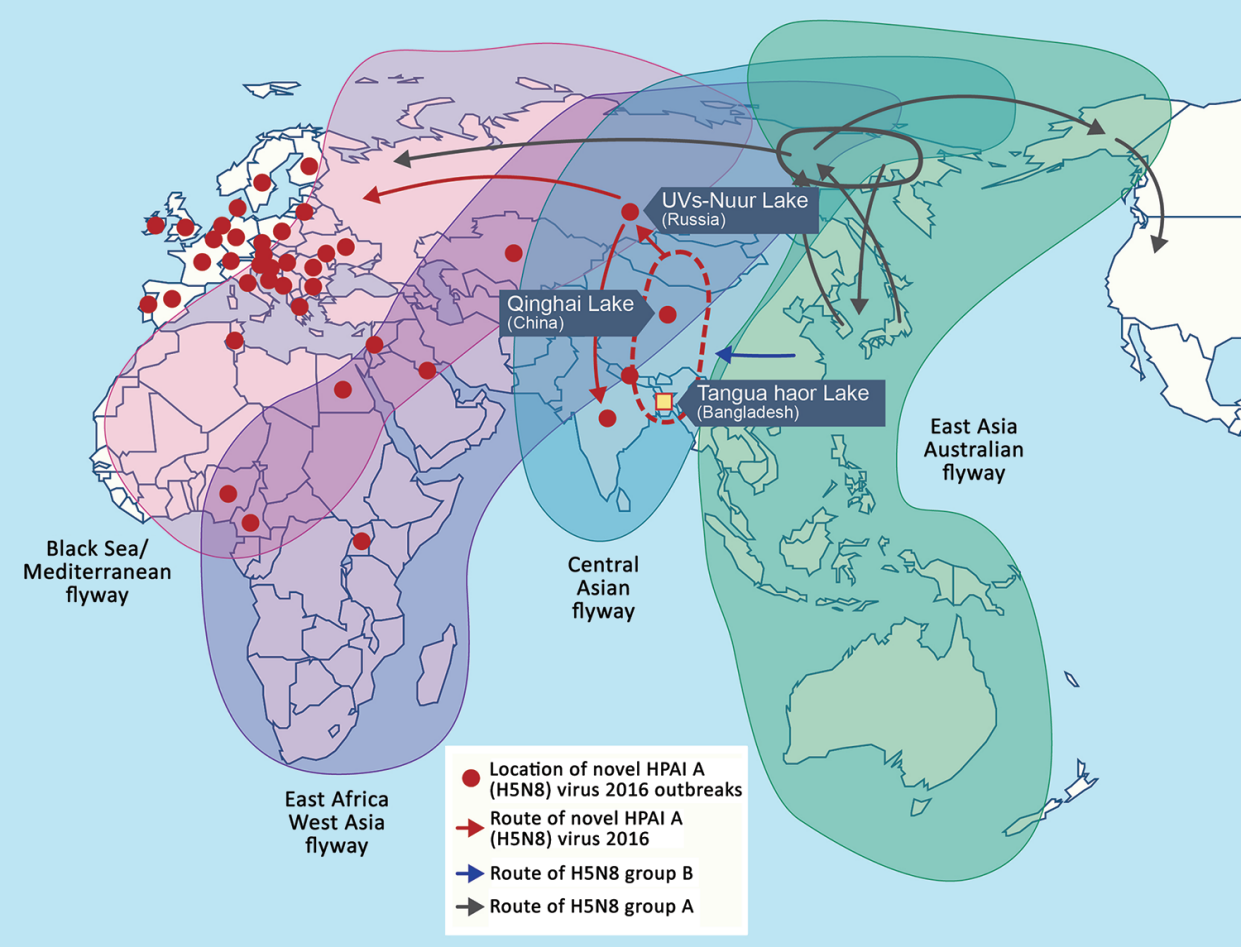
Global movement of wild birds (adapted from [*8*]) and geographic distribution of novel HPAI A(H5N8) viruses, 2016. Influenza A viruses were isolated from wild birds and free-ranging domestic ducks in the Tanguar haor region of Bangladesh (yellow square) during February 2015–February 2016. Dissemination of novel HPAI A(H5N8) clade 2.3.4.4 viruses (red arrows). Dashed circles indicate location of reassortment between HPAI A(H5N8) group B viruses and low pathogenicity avian influenza viruses circulating along the Central Asian flyway. HPAI, highly pathogenic avian influenza.

Since 2008, we have conducted long-term, active surveillance of influenza viruses in poultry in Bangladesh (*7*). From February 2015 through February 2016, we collected samples from wild birds and free-ranging domestic ducks in the Tanguar haor area, a vast wetland in northeastern Bangladesh, where ≈200 types of migratory birds overwinter. Tanguar haor is located in the central Asian flyway and is near the Eastern Asian–Australian and Black Sea–Mediterranean flyways (Figure 1). We collected cloacal swabs from the birds and performed virus isolation and subtyping via reverse transcription PCR (*8*).

During the surveillance period, we isolated 4 influenza A(H3N6), 4 influenza A(H7N1), 1 influenza A(H7N5), 3 influenza A(H7N9), and 2 influenza A(H15N9) viruses, all from free-ranging domestic ducks except for a single H7N5 virus, which was isolated from a migratory black-tailed godwit (Technical Appendix Table). When analyzed individually, gene segments across viruses of different subtypes seem to have evolved closely with viruses from Eurasia. To determine the genetic relatedness between these viruses and the 2016 novel HPAI A(H5N8) virus, we compared our isolates with available sequences of A(H5N8) viruses in GenBank and the GISAID database (http://platform.gisaid.org). We used MEGA6 to generate phylogenetic trees (*9*).

HA, neuraminidase (NA), and nonstructural protein (NS) genes of novel HPAI A(H5N8) viruses were closely related to those of the group B HPAI A(H5N8) viruses that circulated in China in 2013 and in South Korea in 2014 (*5*,*10*). In contrast, the polymerase basic (PB) 2, PB1, polymerase acidic (PA), nucleoprotein (NP), and matrix protein (M) genes of the novel HPAI A(H5N8) viruses were most closely related to those of the LPAI viruses isolated from the central Asia flyway (Technical Appendix Figure).

**Figure 2:**
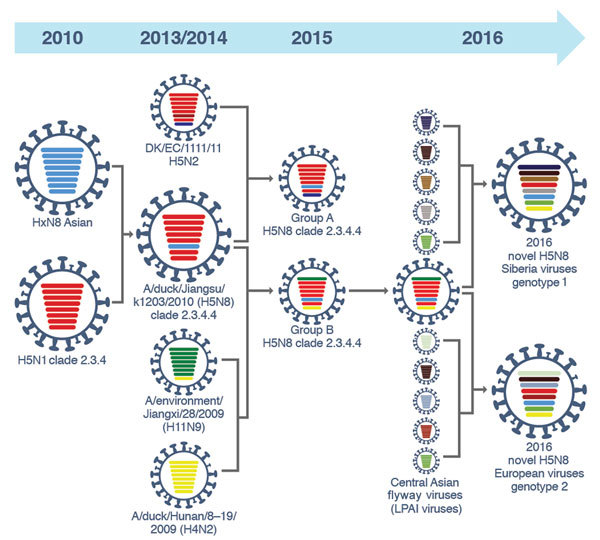
Illustration of original reassortment events of novel highly pathogenic avian influenza (HPAI) A(H5N8) viruses isolated from Siberia and Europe in 2016. The 8 gene segments (from top to bottom) in each virus are polymerase basic 2, polymerase basic 1, polymerase acidic, hemagglutinin, nucleoprotein, neuraminidase, matrix, and nonstructural. Each color indicates a separate virus background. In 2010, HPAI A(H5N1) clade 2.3.4 viruses reassorted with subtype N8 viruses from Eurasia and produced A/duck/Jiangsu/k1203/2010(H5N8). Until late 2013, HPAI viruses with H5N8 subtypes circulated in eastern China and South Korea. In 2014, HPAI A(H5N8) viruses reassorted with A/duck/Hunan/8–19/2009(H4N2) and A/environment/Jiangxi/28/2009(H11N9) to generate group B viruses. The subsequent reassortment between HPAI A(H5N8) group B viruses and low pathogenicity (LPAI) viruses circulating along the central Asian flyway led to generation of the novel HPAI A(H5N8) genotype 1 and 2 viruses.

Sequence analysis of novel HPAI A(H5N8) viruses revealed that sequence similarity of HA, NA, PB1, M, and NS was 99.9%–100%. Sequence homology of PB2, PA, and NP gene segments led to classification of novel HPAI A(H5N8) viruses into 2 genotypes: genotype 1 viruses isolated from Siberia and genotype 2 viruses isolated from Europe (Figure 2).

To explore the possible genetic exchange between LPAI viruses isolated from the Tanguar haor area and novel HPAI A(H5N8) viruses, we analyzed the phylogeny and nucleotide identity of the M gene and internal gene sequences (Technical Appendix). The PB2 genes of HPAI A(H5N8) genotype 1 viruses were closely related to those of the influenza A(H4N6) virus strain from Mongolia and shared identity homology with 3 influenza A(H7N1) viruses; sequence identities ranged from 98.1% to 98.6%. Genotype 2 viruses were related to influenza A(H3N6) viruses; identities were 98.6%–98.9%. The PB1 genes of HPAI A(H5N8) genotype 1 and 2 viruses were related to those of A/duck/Bangladesh/26918/2015(H3N6); identities were 97.3%–98.0% (Table). The PA genes of genotype 1 viruses were more closely related to those of the Mongolia strains of influenza A(H3N8) and A(H4N6) viruses. Genotype 2 viruses were more closely related to those of A/duck/Bangladesh/26918/2015(H3N6); identities were 97.1%–97.3%. The NP genes of genotype 1 viruses were more closely related to those of influenza A(H7N9) viruses; identities were 98.4%–98.6%. However, the NP genes of genotype 2 viruses were more closely related to those of influenza A(H3N6) and A(H7N1) viruses; identities were 97%–97.2%. The M genes of genotypes 1 and 2 viruses were related to those of influenza A(H15N9) viruses; identities were 98%–98.5% (Table).

**Table:** Nucleotide identity of novel HPAI A(H5N8) clade 2.3.4.4 virus and viruses isolated from Tanguar haor, Bangladesh*

Gene and genotype, novel HPAI A(H5N8) clade 2.3.4.4, 2016	Viruses from Tanguar haor (Central Asian flyway)†	% Identity
PB2		
Genotype 1‡	A/duck/Bangladesh/24705/2015(H7N1)§	98.4–98.6
Genotype 2‡	A/duck/Bangladesh/26920/2015(H3N6)	98.7–98.9
PB1	A/duck/Bangladesh/26918/2015(H3N6)	97.3–98
PA		
Genotype 1‡	A/duck/Bangladesh/24706/2015(H7N1)	95.3
Genotype 2‡	A/duck/Bangladesh/26918/2015(H3N6)	97.1–97.3
NP		
Genotype 1‡	A/duck/Bangladesh/26992/2015(H7N9)	98.6
Genotype 2‡	A/duck/Bangladesh/24706/2015(H7N1)	97–97.1
M	A/duck/Bangladesh/24704/2015(H15N9)	98–98.5

*HPAI, highly pathogenic avian influenza; NP, nucleoprotein; M, matrix; PA, polymerase acidic; PB, polymerase basic.  †All Eurasian low-pathogenicity avian influenza lineage. ‡Genotype 1 viruses isolated from Siberia; genotype 2 viruses isolated from Europe. §Selected 1 representative virus isolate from the Tanguar haor region of Bangladesh.

We next determined the presence of genetic markers associated with pathogenicity and virulence in mammals or adaptation to new hosts. On the basis of the amino acids at positions 591, 627, and 701 in the PB2 protein, the viruses are likely to exhibit low pathogenicity in mice. However, NS residues P42S and V149A, associated with virulence and pathogenicity in mammals, were in all Tanguar haor isolates and HPAI A(H5N8) viruses (*11*,*12*).

## Conclusions

In 2016, a novel HPAI A(H5N8) virus clade 2.3.4.4 emerged and spread to Russia, Europe, and Africa. We demonstrated that several internal genes from viruses in ducks in Bangladesh have an equivalent or higher consensus identity to those of other viruses of wild birds in central Asia, suggesting that these viruses could be gene donors to the novel reassortant A(H5N8) viruses, which were then disseminated by wild birds. The novel HPAI A(H5N8) viruses diverged along 2 genotypes with independent origins of reassortment for several gene segments. The HA, NA, and NS genes were related to group B of H5N8 clade 2.3.4.4 viruses that circulated in China from 2013. Group B is still circulating in China, and a previous study showed that these viruses had PB2 and NS genes derived from domestic ducks in eastern China (*13*), indicating further reassortment events.

The route of spread of HPAI A(H5N1) viruses from eastern Asia to Europe, Africa, and the Middle East in 2005 and 2006 most likely occurred by spillover infection from wild birds. HPAI A(H5N1) viruses were detected during an outbreak among migratory birds at Qinghai Lake in China, which is located in the central Asian flyway (*14*), suggesting that this flyway is a route for dissemination of HPAI A(H5N1) viruses. A recent study suggested that only the PA and NP segments of 2016 A(H5N8) viruses isolated in Germany differed from those of genotype 1 viruses isolated in Siberia, suggesting that reassortment occurred with viruses circulating in central Asia and northwestern Europe (*10*). However, we show that the PB2, PA, and NP genes of genotype 1 viruses not only differed from those of genotype 2 viruses but clustered with and were more closely related to those of viruses from Bangladesh and central Asia. Active surveillance of influenza viruses among migratory wild birds and molecular studies need to be sustained to monitor the spread of these viruses through wild birds.

Technical Appendix Influenza viruses isolated from wild birds and free-ranging domestic ducks in the Tanguar haor region of Bangladesh, February 2015–February 2016, and phylogenetic trees for the genes of these viruses.
