# Supplementary material for: Genesis of Influenza A(H5N8) Viruses
Source: Emerg Infect Dis. 2017 Aug;23(8):1368–71. doi: 10.3201/eid2308.170143 (PMC5547793; doi:10.3201/eid2308.170143)
Supplement: Technical Appendix — Influenza viruses isolated from wild birds and free-ranging domestic ducks in the Tanguar haor region of Bangladesh, February 2015–February 2016, and phylogenetic trees for the genes of these viruses. [file 17-0143-Techapp-s1.pdf]

Article DOI: https://doi.org/10.3201/eid2308.170143

Genesis of Influenza A(H5N8) Viruses

Technical Appendix

Technical Appendix Table. Summary of influenza viruses isolated from the Tanguar haor region of Bangladesh*

Isolates	Subtype	Host (Species)	GenBank Accession Numbers							
			PB2	PB1	PA	HA	NP	NA	M	NS
A/duck/Bangladesh/24692/2015	H7N1	domestic duck (*Anas sp.*)	KY635738	KY635673	KY635658	KY635574	KY635806	KY635787	KY635810	KY635768
A/duck/Bangladesh/24694/2015	H7N1	domestic duck (*Anas sp.*)	KY635469	KY635519	KY635624	KY635530	KY635523	KY635813	KY635603	KY635444
A/duck/Bangladesh/24697/2015	H15N9	domestic duck (*Anas sp.*)	KY635497	KY635750	KY635663	KY635719	KY635528	KY635723	KY635819	KY635715
A/duck/Bangladesh/24704/2015	H15N9	domestic duck (*Anas sp.*)	KY635570	KY635825	KY635563	KY635680	KY635694	KY635500	KY635683	KY635679
A/duck/Bangladesh/24705/2015	H7N1	domestic duck (*Anas sp.*)	KY635677	ND†	KY635807	KY635675	KY635484	KY635654	KY635804	KY635493
A/duck/Bangladesh/24706/2015	H7N1	domestic duck (*Anas sp.*)	KY635502	KY635462	KY635447	KY635584	KY635698	KY635443	KY635562	KY635446
A/black-tailed godwit/Bangladesh/24734/2015	H7N5	black-tailed godwit (*Limosa limosa*)	KY635643	KY635517	KY635496	KY635587	KY635709	KY635758	KY635591	KY635798
A/duck/Bangladesh/26918/2015	H3N6	domestic duck (*Anas sp.*)	KY635524	KY635797	KY635609	KY635482	KY635636	KY635579	KY635602	KY635490
A/duck/Bangladesh/26920/2015	H3N6	domestic duck (*Anas sp.*)	KY635499	KY635617	KY635731	KY635779	KY635705	KY635782	KY635634	KY635478
A/duck/Bangladesh/26948/2015	H3N6	domestic duck (*Anas sp.*)	KY635545	KY635604	KY635655	KY635661	KY635745	KY635653	KY635507	KY635792
A/duck/Bangladesh/26974/2015	H3N6	domestic duck (*Anas sp.*)	KY635593	KY635626	KY635597	KY635571	KY635639	KY635735	KY635720	KY635520
A/duck/Bangladesh/26980/2015	H7N9	domestic duck (*Anas sp.*)	KY635459	KY635811	KY635690	KY635734	KY635442	KY635689	KY635666	KY635659
A/duck/Bangladesh/26992/2015	H7N9	domestic duck (*Anas sp.*)	KY635525	KY635621	KY635802	KY635780	KY635633	KY635541	KY635753	KY635550
A/duck/Bangladesh/27042/2015	H7N9	domestic duck (*Anas sp.*)	KY635516	KY635641	KY635827	KY635561	KY635733	KY635739	KY635509	KY635772

*ND, not done.

Technical Appendix Figure (following pages). Phylogenetic trees for the (A) polymerase basic-2 (PB2), (B) polymerase basic-1 (PB1), (C) polymerase acidic (PA), (D) nucleoprotein (NP), (E) matrix (M), and (F) nonstructural (NS) genes of viruses isolated from the Tanguar haor area in Bangladesh (blue font) and HPAI A(H5N8) clade 2.3.4.4, 2016 viruses (red font). Phylogenetic analysis was performed with the neighbor-joining algorithm with the Kimura 2-parameter model. The reliability of the phylogenetic inference at each branch node was estimated by the bootstrap method with 1,000 replications. Evolutionary analyses were conducted with MEGA6 software.

A PB2

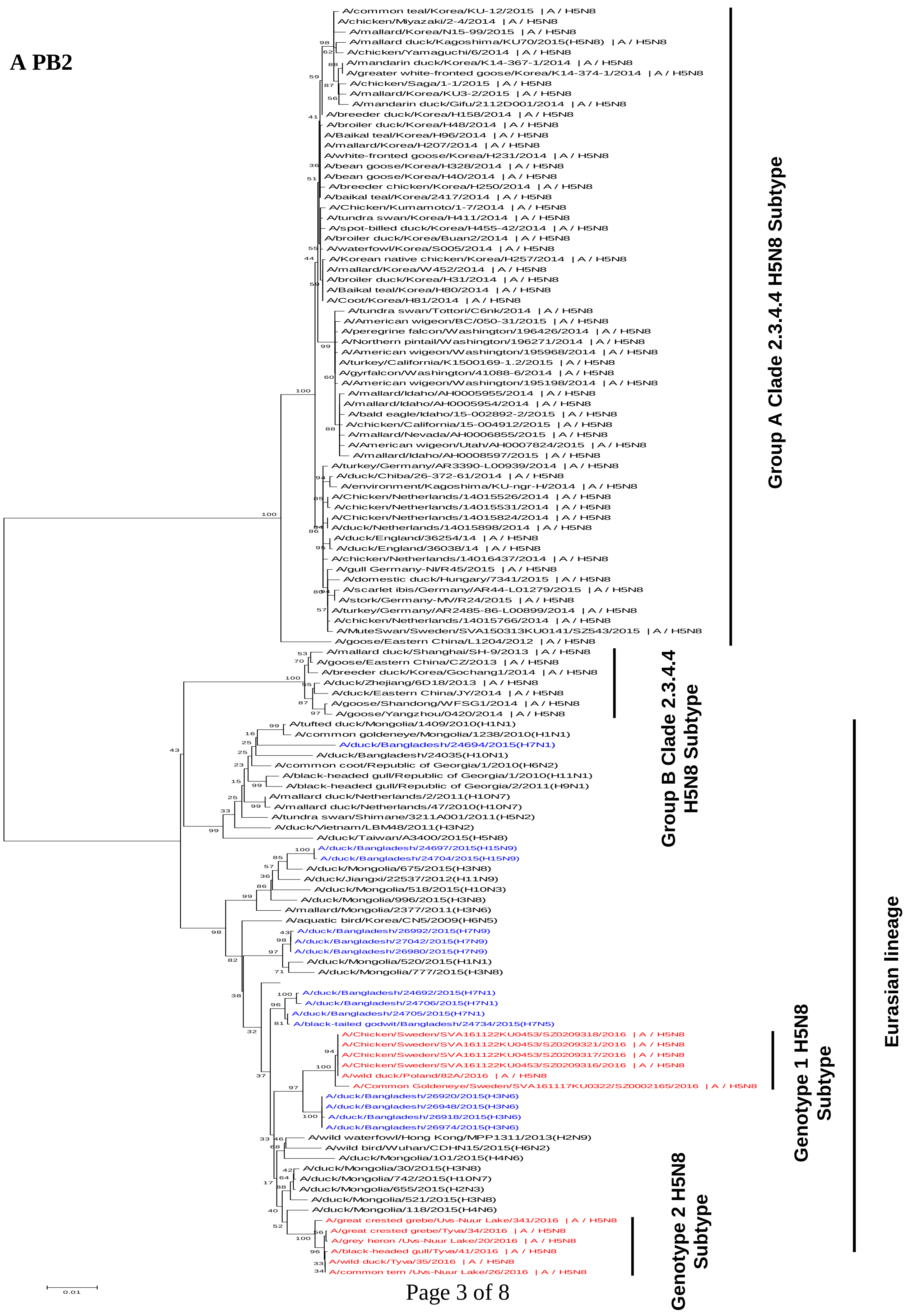

A/common teal/Korea/KU-12/2015 | A / H5N8
A/chicken/Miyazaki/2-4/2014 | A / H5N8
A/mallard/Korea/N15-99/2015 | A / H5N8
A/mallard duck/Kagoshima/KU70/2015(H5N8) | A / H5N8
A/chicken/Yamaguchi/6/2014 | A / H5N8
A/mandarin duck/Korea/K14-367-1/2014 | A / H5N8
A/greater white-fronted goose/Korea/K14-374-1/2014 | A / H5N8
A/chicken/Saga/1-1/2015 | A / H5N8
A/mallard/Korea/KU3-2/2015 | A / H5N8
A/mandarin duck/Gifu/2112D001/2014 | A / H5N8
A/breeder duck/Korea/H158/2014 | A / H5N8
A/broiler duck/Korea/H48/2014 | A / H5N8
A/Baikal teal/Korea/H96/2014 | A / H5N8
A/mallard/Korea/H207/2014 | A / H5N8
A/white-fronted goose/Korea/H231/2014 | A / H5N8
A/bean goose/Korea/H328/2014 | A / H5N8
A/bean goose/Korea/H40/2014 | A / H5N8
A/breeder chicken/Korea/H250/2014 | A / H5N8
A/baikal teal/Korea/2417/2014 | A / H5N8
A/Chicken/Kumamoto/1-7/2014 | A / H5N8
A/tundra swan/Korea/H411/2014 | A / H5N8
A/spot-billed duck/Korea/H455-42/2014 | A / H5N8
A/broiler duck/Korea/Buan2/2014 | A / H5N8
A/waterfowl/Korea/S005/2014 | A / H5N8
A/Korean native chicken/Korea/H257/2014 | A / H5N8
A/mallard/Korea/W452/2014 | A / H5N8
A/broiler duck/Korea/H31/2014 | A / H5N8
A/Baikal teal/Korea/H80/2014 | A / H5N8
A/Coot/Korea/H81/2014 | A / H5N8
A/tundra swan/Tottori/C6nk/2014 | A / H5N8
A/American wigeon/BC/050-31/2015 | A / H5N8
A/peregrine falcon/Washington/196426/2014 | A / H5N8
A/Northern pintail/Washington/196271/2014 | A / H5N8
A/American wigeon/Washington/195968/2014 | A / H5N8
A/turkey/California/K1500169-1.2/2015 | A / H5N8
A/gyrfalcon/Washington/41088-6/2014 | A / H5N8
A/American wigeon/Washington/195198/2014 | A / H5N8
A/mallard/Idaho/AH0005955/2014 | A / H5N8
A/mallard/Idaho/AH0005954/2014 | A / H5N8
A/bald eagle/Idaho/15-002892-2/2015 | A / H5N8
A/chicken/California/15-004912/2015 | A / H5N8
A/mallard/Nevada/AH0006855/2015 | A / H5N8
A/American wigeon/Utah/AH0007824/2015 | A / H5N8
A/mallard/Idaho/AH0008597/2015 | A / H5N8
A/turkey/Germany/AR3390-L00939/2014 | A / H5N8
A/duck/Chiba/26-372-61/2014 | A / H5N8
A/environment/Kagoshima/KU-ngr-H/2014 | A / H5N8
A/Chicken/Netherlands/14015526/2014 | A / H5N8
A/chicken/Netherlands/14015531/2014 | A / H5N8
A/Chicken/Netherlands/14015824/2014 | A / H5N8
A/duck/Netherlands/14015898/2014 | A / H5N8
A/duck/England/36254/14 | A / H5N8
A/duck/England/36038/14 | A / H5N8
A/chicken/Netherlands/14016437/2014 | A / H5N8
A/gull Germany-NI/R45/2015 | A / H5N8
A/domestic duck/Hungary/7341/2015 | A / H5N8
A/scarlet ibis/Germany/AR44-L01279/2015 | A / H5N8
A/stork/Germany-MV/R24/2015 | A / H5N8
A/turkey/Germany/AR2485-86-L00899/2014 | A / H5N8
A/chicken/Netherlands/14015766/2014 | A / H5N8
A/MuteSwan/Sweden/SVA150313KU0141/SZ543/2015 | A / H5N8
A/goose/Eastern China/L1204/2012 | A / H5N8
A/mallard duck/Shanghai/SH-9/2013 | A / H5N8
A/goose/Eastern China/CZ/2013 | A / H5N8
A/breeder duck/Korea/Gochang1/2014 | A / H5N8
A/duck/Zhejiang/6D18/2013 | A / H5N8
A/duck/Eastern China/JY/2014 | A / H5N8
A/goose/Shandong/WFSG1/2014 | A / H5N8
A/goose/Yangzhou/0420/2014 | A / H5N8
A/tufted duck/Mongolia/1409/2010(H1N1)
A/common goldeneye/Mongolia/1238/2010(H1N1)
A/duck/Bangladesh/24694/2015(H7N1)
A/duck/Bangladesh/24035(H10N1)
A/common coot/Republic of Georgia/1/2010(H6N2)
A/black-headed gull/Republic of Georgia/1/2010(H11N1)
A/black-headed gull/Republic of Georgia/2/2011(H9N1)
A/mallard duck/Netherlands/2/2011(H10N7)
A/mallard duck/Netherlands/47/2010(H10N7)
A/tundra swan/Shimane/3211A001/2011(H5N2)
A/duck/Vietnam/LBM48/2011(H3N2)
A/duck/Taiwan/A3400/2015(H5N8)
A/duck/Bangladesh/24697/2015(H15N9)
A/duck/Bangladesh/24704/2015(H15N9)
A/duck/Mongolia/675/2015(H3N8)
A/duck/Jiangxi/22537/2012(H11N9)
A/duck/Mongolia/518/2015(H10N3)
A/duck/Mongolia/996/2015(H3N8)
A/mallard/Mongolia/2377/2011(H3N6)
A/aquatic bird/Korea/CN5/2009(H6N5)
A/duck/Bangladesh/26992/2015(H7N9)
A/duck/Bangladesh/27042/2015(H7N9)
A/duck/Bangladesh/26980/2015(H7N9)
A/duck/Mongolia/520/2015(H1N1)
A/duck/Mongolia/777/2015(H3N8)
A/duck/Bangladesh/24692/2015(H7N1)
A/duck/Bangladesh/24706/2015(H7N1)
A/duck/Bangladesh/24705/2015(H7N1)
A/black-tailed godwit/Bangladesh/24734/2015(H7N5)
A/Chicken/Sweden/SVA161122KU0453/SZ0209318/2016 | A / H5N8
A/Chicken/Sweden/SVA161122KU0453/SZ0209321/2016 | A / H5N8
A/Chicken/Sweden/SVA161122KU0453/SZ0209317/2016 | A / H5N8
A/Chicken/Sweden/SVA161122KU0453/SZ0209316/2016 | A / H5N8
A/wild duck/Poland/82A/2016 | A / H5N8
A/Common Goldeneye/Sweden/SVA161117KU0322/SZ0002165/2016 | A / H5N8
A/duck/Bangladesh/26920/2015(H3N6)
A/duck/Bangladesh/26948/2015(H3N6)
A/duck/Bangladesh/26918/2015(H3N6)
A/duck/Bangladesh/26974/2015(H3N6)
A/wild waterfowl/Hong Kong/MPP1311/2013(H2N9)
A/wild bird/Wuhan/CDHN15/2015(H6N2)
A/duck/Mongolia/101/2015(H4N6)
A/duck/Mongolia/30/2015(H3N8)
A/duck/Mongolia/742/2015(H10N7)
A/duck/Mongolia/655/2015(H2N3)
A/duck/Mongolia/521/2015(H3N8)
A/duck/Mongolia/118/2015(H4N6)
A/great crested grebe/Uvs-Nuur Lake/341/2016 | A / H5N8
A/great crested grebe/Tyva/34/2016 | A / H5N8
A/grey heron /Uvs-Nuur Lake/20/2016 | A / H5N8
A/black-headed gull/Tyva/41/2016 | A / H5N8
A/wild duck/Tyva/35/2016 | A / H5N8
A/common tern /Uvs-Nuur Lake/26/2016 | A / H5N8
Group A Clade 2.3.4.4 H5N8 Subtype
Group B Clade 2.3.4.4 H5N8 Subtype
Genotype 1 H5N8 Subtype
Genotype 2 H5N8 Subtype
Eurasian lineage
0.01

B PB1

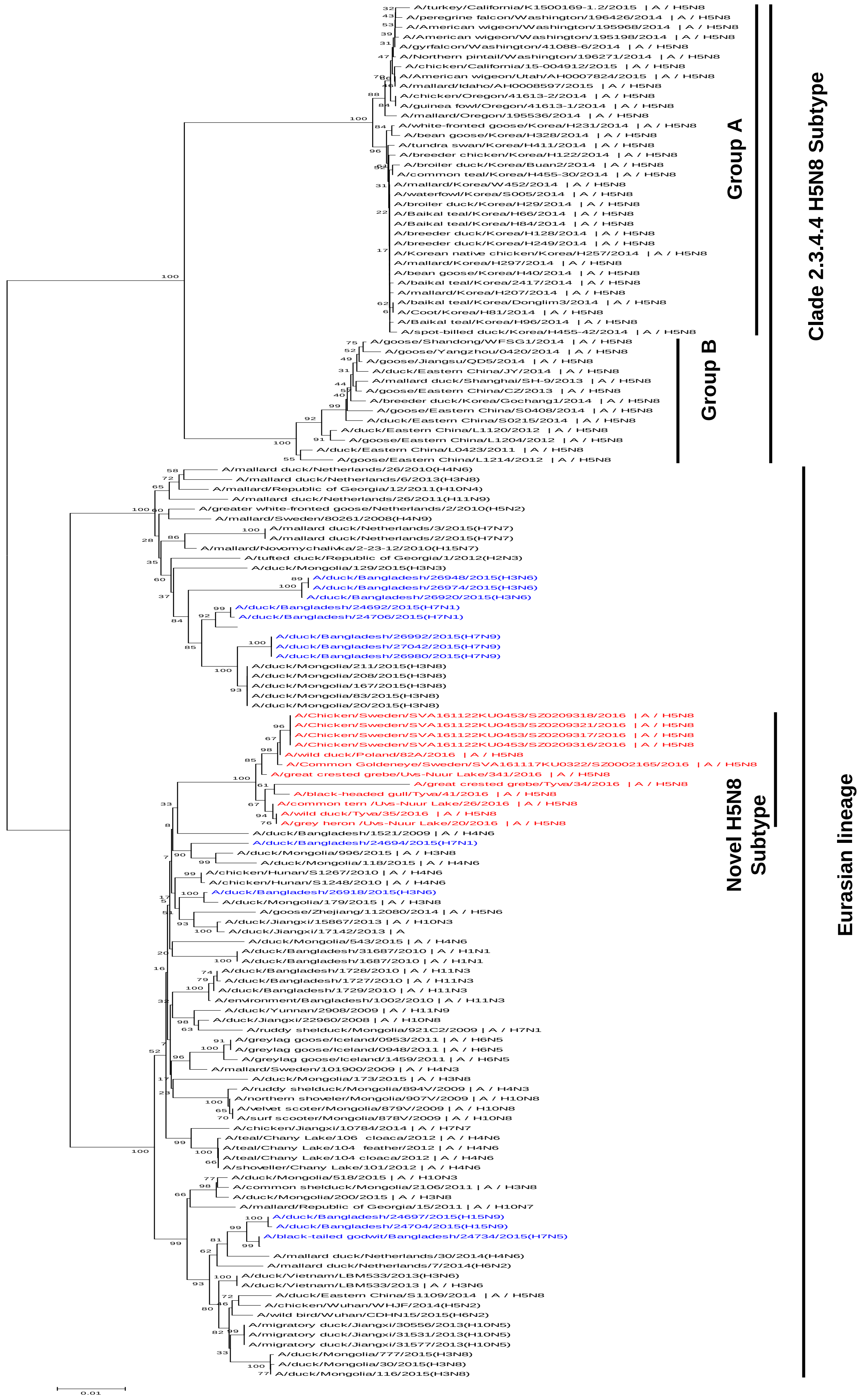
A/turkey/California/K1500169-1.2/2015 | A / H5N8
A/peregrine falcon/Washington/196426/2014 | A / H5N8
A/American wigeon/Washington/195968/2014 | A / H5N8
A/American wigeon/Washington/195198/2014 | A / H5N8
A/gyrfalcon/Washington/41088-6/2014 | A / H5N8
A/Northern pintail/Washington/196271/2014 | A / H5N8
A/chicken/California/15-004912/2015 | A / H5N8
A/American wigeon/Utah/AH0007824/2015 | A / H5N8
A/mallard/Idaho/AH0008597/2015 | A / H5N8
A/chicken/Oregon/41613-2/2014 | A / H5N8
A/guinea fowl/Oregon/41613-1/2014 | A / H5N8
A/mallard/Oregon/195536/2014 | A / H5N8
A/white-fronted goose/Korea/H231/2014 | A / H5N8
A/bean goose/Korea/H328/2014 | A / H5N8
A/tundra swan/Korea/H411/2014 | A / H5N8
A/breeder chicken/Korea/H122/2014 | A / H5N8
A/broiler duck/Korea/Buan2/2014 | A / H5N8
A/common teal/Korea/H455-30/2014 | A / H5N8
A/mallard/Korea/W452/2014 | A / H5N8
A/waterfowl/Korea/S005/2014 | A / H5N8
A/broiler duck/Korea/H29/2014 | A / H5N8
A/Baikal teal/Korea/H66/2014 | A / H5N8
A/Baikal teal/Korea/H84/2014 | A / H5N8
A/breeder duck/Korea/H128/2014 | A / H5N8
A/breeder duck/Korea/H249/2014 | A / H5N8
A/Korean native chicken/Korea/H257/2014 | A / H5N8
A/mallard/Korea/H297/2014 | A / H5N8
A/bean goose/Korea/H40/2014 | A / H5N8
A/baikal teal/Korea/2417/2014 | A / H5N8
A/mallard/Korea/H207/2014 | A / H5N8
A/baikal teal/Korea/Donglim3/2014 | A / H5N8
A/Coot/Korea/H81/2014 | A / H5N8
A/Baikal teal/Korea/H96/2014 | A / H5N8
A/spot-billed duck/Korea/H455-42/2014 | A / H5N8
A/goose/Shandong/WFSG1/2014 | A / H5N8
A/goose/Yangzhou/0420/2014 | A / H5N8
A/goose/Jiangsu/QD5/2014 | A / H5N8
A/duck/Eastern China/JY/2014 | A / H5N8
A/mallard duck/Shanghai/SH-9/2013 | A / H5N8
A/goose/Eastern China/CZ/2013 | A / H5N8
A/breeder duck/Korea/Gochang1/2014 | A / H5N8
A/goose/Eastern China/S0408/2014 | A / H5N8
A/duck/Eastern China/S0215/2014 | A / H5N8
A/duck/Eastern China/L1120/2012 | A / H5N8
A/goose/Eastern China/L1204/2012 | A / H5N8
A/duck/Eastern China/L0423/2011 | A / H5N8
A/goose/Eastern China/L1214/2012 | A / H5N8
A/mallard duck/Netherlands/26/2010(H4N6)
A/mallard duck/Netherlands/6/2013(H3N8)
A/mallard/Republic of Georgia/12/2011(H10N4)
A/mallard duck/Netherlands/26/2011(H11N9)
A/greater white-fronted goose/Netherlands/2/2010(H5N2)
A/mallard/Sweden/80261/2008(H4N9)
A/mallard duck/Netherlands/3/2015(H7N7)
A/mallard duck/Netherlands/2/2015(H7N7)
A/mallard/Novomychalivka/2-23-12/2010(H15N7)
A/tufted duck/Republic of Georgia/1/2012(H2N3)
A/duck/Mongolia/129/2015(H3N3)
A/duck/Bangladesh/26948/2015(H3N6)
A/duck/Bangladesh/26974/2015(H3N6)
A/duck/Bangladesh/26920/2015(H3N6)
A/duck/Bangladesh/24692/2015(H7N1)
A/duck/Bangladesh/24706/2015(H7N1)
A/duck/Bangladesh/26992/2015(H7N9)
A/duck/Bangladesh/27042/2015(H7N9)
A/duck/Bangladesh/26980/2015(H7N9)
A/duck/Mongolia/211/2015(H3N8)
A/duck/Mongolia/208/2015(H3N8)
A/duck/Mongolia/167/2015(H3N8)
A/duck/Mongolia/83/2015(H3N8)
A/duck/Mongolia/20/2015(H3N8)
A/Chicken/Sweden/SVA161122KU0453/SZ0209318/2016 | A / H5N8
A/Chicken/Sweden/SVA161122KU0453/SZ0209321/2016 | A / H5N8
A/Chicken/Sweden/SVA161122KU0453/SZ0209317/2016 | A / H5N8
A/Chicken/Sweden/SVA161122KU0453/SZ0209316/2016 | A / H5N8
A/wild duck/Poland/82A/2016 | A / H5N8
A/Common Goldeneye/Sweden/SVA161117KU0322/SZ0002165/2016 | A / H5N8
A/great crested grebe/Uvs-Nuur Lake/341/2016 | A / H5N8
A/great crested grebe/Tyva/34/2016 | A / H5N8
A/black-headed gull/Tyva/41/2016 | A / H5N8
A/common tern /Uvs-Nuur Lake/26/2016 | A / H5N8
A/wild duck/Tyva/35/2016 | A / H5N8
A/grey heron /Uvs-Nuur Lake/20/2016 | A / H5N8
A/duck/Bangladesh/1521/2009 | A / H4N6
A/duck/Bangladesh/24694/2015(H7N1)
A/duck/Mongolia/996/2015 | A / H3N8
A/duck/Mongolia/118/2015 | A / H4N6
A/chicken/Hunan/S1267/2010 | A / H4N6
A/chicken/Hunan/S1248/2010 | A / H4N6
A/duck/Bangladesh/26918/2015(H3N6)
A/duck/Mongolia/179/2015 | A / H3N8
A/goose/Zhejiang/112080/2014 | A / H5N6
A/duck/Jiangxi/15867/2013 | A / H10N3
A/duck/Jiangxi/17142/2013 | A
A/duck/Mongolia/543/2015 | A / H4N6
A/duck/Bangladesh/31687/2010 | A / H1N1
A/duck/Bangladesh/1687/2010 | A / H1N1
A/duck/Bangladesh/1728/2010 | A / H11N3
A/duck/Bangladesh/1727/2010 | A / H11N3
A/duck/Bangladesh/1729/2010 | A / H11N3
A/environment/Bangladesh/1002/2010 | A / H11N3
A/duck/Yunnan/2908/2009 | A / H11N9
A/duck/Jiangxi/22960/2008 | A / H10N8
A/ruddy shelduck/Mongolia/921C2/2009 | A / H7N1
A/greylag goose/Iceland/0953/2011 | A / H6N5
A/greylag goose/Iceland/0948/2011 | A / H6N5
A/greylag goose/Iceland/1459/2011 | A / H6N5
A/mallard/Sweden/101900/2009 | A / H4N3
A/duck/Mongolia/173/2015 | A / H3N8
A/ruddy shelduck/Mongolia/894V/2009 | A / H4N3
A/northern shoveler/Mongolia/907V/2009 | A / H10N8
A/velvet scoter/Mongolia/879V/2009 | A / H10N8
A/surf scooter/Mongolia/878V/2009 | A / H10N8
A/chicken/Jiangxi/10784/2014 | A / H7N7
A/teal/Chany Lake/106 cloaca/2012 | A / H4N6
A/teal/Chany Lake/104 feather/2012 | A / H4N6
A/teal/Chany Lake/104 cloaca/2012 | A / H4N6
A/shoveller/Chany Lake/101/2012 | A / H4N6
A/duck/Mongolia/518/2015 | A / H10N3
A/common shelduck/Mongolia/2106/2011 | A / H3N8
A/duck/Mongolia/200/2015 | A / H3N8
A/mallard/Republic of Georgia/15/2011 | A / H10N7
A/duck/Bangladesh/24697/2015(H15N9)
A/duck/Bangladesh/24704/2015(H15N9)
A/black-tailed godwit/Bangladesh/24734/2015(H7N5)
A/mallard duck/Netherlands/30/2014(H4N6)
A/mallard duck/Netherlands/7/2014(H6N2)
A/duck/Vietnam/LBM533/2013(H3N6)
A/duck/Vietnam/LBM533/2013 | A / H3N6
A/duck/Eastern China/S1109/2014 | A / H5N8
A/chicken/Wuhan/WHJF/2014(H5N2)
A/wild bird/Wuhan/CDHN15/2015(H6N2)
A/migratory duck/Jiangxi/30556/2013(H10N5)
A/migratory duck/Jiangxi/31531/2013(H10N5)
A/migratory duck/Jiangxi/31577/2013(H10N5)
A/duck/Mongolia/777/2015(H3N8)
A/duck/Mongolia/30/2015(H3N8)
A/duck/Mongolia/116/2015(H3N8)
0.01
Group A
Group B
Clade 2.3.4.4 H5N8 Subtype
Novel H5N8 Subtype
Eurasian lineage

C PA

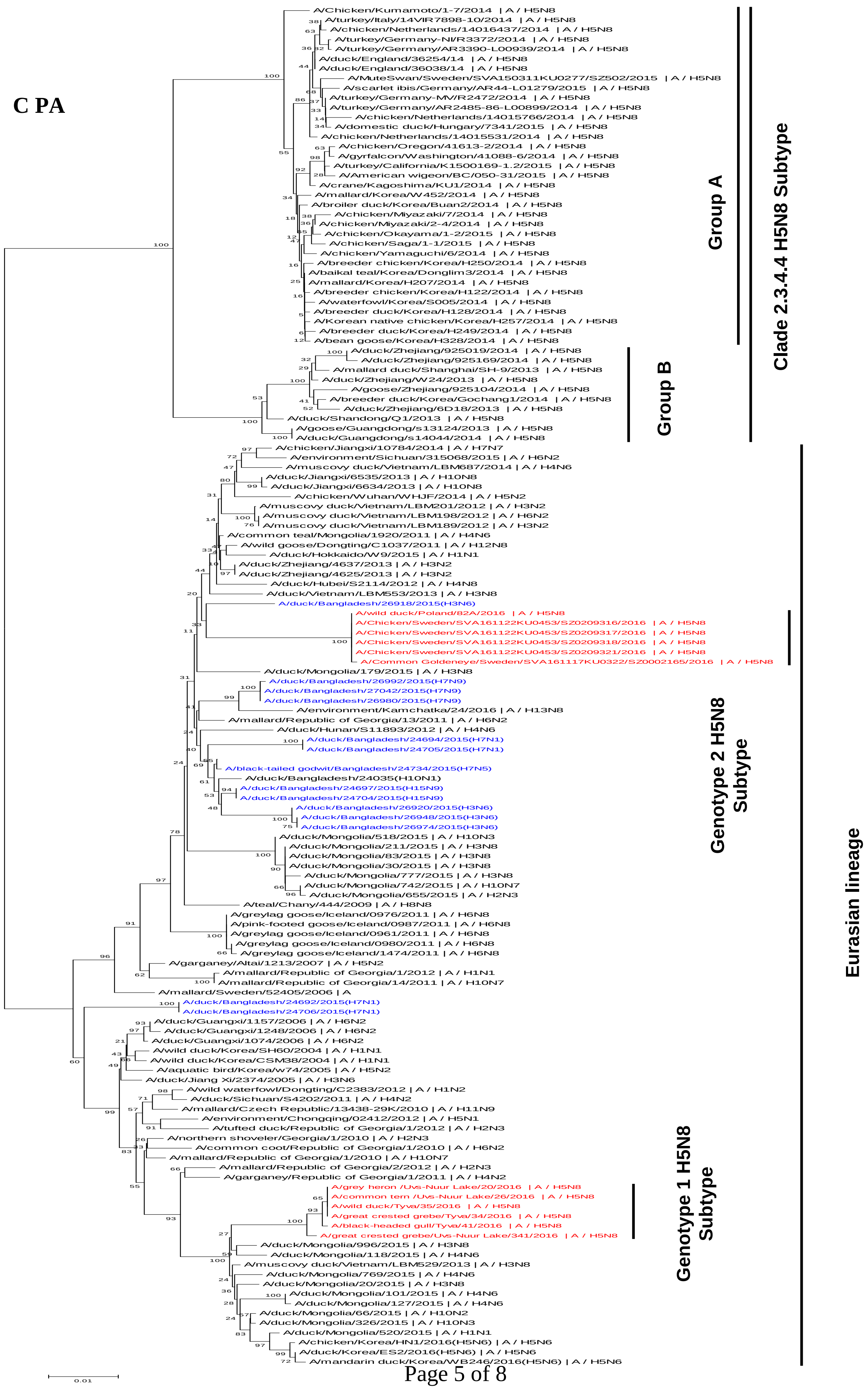
A/Chicken/Kumamoto/1-7/2014 | A / H5N8
A/turkey/Italy/14VIR7898-10/2014 | A / H5N8
A/chicken/Netherlands/14016437/2014 | A / H5N8
A/turkey/Germany-NI/R3372/2014 | A / H5N8
A/turkey/Germany/AR3390-L00939/2014 | A / H5N8
A/duck/England/36254/14 | A / H5N8
A/duck/England/36038/14 | A / H5N8
A/MuteSwan/Sweden/SVA150311KU0277/SZ502/2015 | A / H5N8
A/scarlet ibis/Germany/AR44-L01279/2015 | A / H5N8
A/turkey/Germany-MV/R2472/2014 | A / H5N8
A/turkey/Germany/AR2485-86-L00899/2014 | A / H5N8
A/chicken/Netherlands/14015766/2014 | A / H5N8
A/domestic duck/Hungary/7341/2015 | A / H5N8
A/chicken/Netherlands/14015531/2014 | A / H5N8
A/chicken/Oregon/41613-2/2014 | A / H5N8
A/gyrfalcon/Washington/41088-6/2014 | A / H5N8
A/turkey/California/K1500169-1.2/2015 | A / H5N8
A/American wigeon/BC/050-31/2015 | A / H5N8
A/crane/Kagoshima/KU1/2014 | A / H5N8
A/mallard/Korea/W452/2014 | A / H5N8
A/broiler duck/Korea/Buan2/2014 | A / H5N8
A/chicken/Miyazaki/7/2014 | A / H5N8
A/chicken/Miyazaki/2-4/2014 | A / H5N8
A/chicken/Okayama/1-2/2015 | A / H5N8
A/chicken/Saga/1-1/2015 | A / H5N8
A/chicken/Yamaguchi/6/2014 | A / H5N8
A/breeder chicken/Korea/H250/2014 | A / H5N8
A/baikal teal/Korea/Donglim3/2014 | A / H5N8
A/mallard/Korea/H207/2014 | A / H5N8
A/breeder chicken/Korea/H122/2014 | A / H5N8
A/waterfowl/Korea/S005/2014 | A / H5N8
A/breeder duck/Korea/H128/2014 | A / H5N8
A/Korean native chicken/Korea/H257/2014 | A / H5N8
A/breeder duck/Korea/H249/2014 | A / H5N8
A/bean goose/Korea/H328/2014 | A / H5N8
A/duck/Zhejiang/925019/2014 | A / H5N8
A/duck/Zhejiang/925169/2014 | A / H5N8
A/mallard duck/Shanghai/SH-9/2013 | A / H5N8
A/duck/Zhejiang/W24/2013 | A / H5N8
A/goose/Zhejiang/925104/2014 | A / H5N8
A/breeder duck/Korea/Gochang1/2014 | A / H5N8
A/duck/Zhejiang/6D18/2013 | A / H5N8
A/duck/Shandong/Q1/2013 | A / H5N8
A/goose/Guangdong/s13124/2013 | A / H5N8
A/duck/Guangdong/s14044/2014 | A / H5N8
A/chicken/Jiangxi/10784/2014 | A / H7N7
A/environment/Sichuan/315068/2015 | A / H6N2
A/muscovy duck/Vietnam/LBM687/2014 | A / H4N6
A/duck/Jiangxi/6535/2013 | A / H10N8
A/duck/Jiangxi/6634/2013 | A / H10N8
A/chicken/Wuhan/WHJF/2014 | A / H5N2
A/muscovy duck/Vietnam/LBM201/2012 | A / H3N2
A/muscovy duck/Vietnam/LBM198/2012 | A / H6N2
A/muscovy duck/Vietnam/LBM189/2012 | A / H3N2
A/common teal/Mongolia/1920/2011 | A / H4N6
A/wild goose/Dongting/C1037/2011 | A / H12N8
A/duck/Hokkaido/W9/2015 | A / H1N1
A/duck/Zhejiang/4637/2013 | A / H3N2
A/duck/Zhejiang/4625/2013 | A / H3N2
A/duck/Hubei/S2114/2012 | A / H4N8
A/duck/Vietnam/LBM553/2013 | A / H3N8
A/duck/Bangladesh/26918/2015(H3N6)
A/wild duck/Poland/82A/2016 | A / H5N8
A/Chicken/Sweden/SVA161122KU0453/SZ0209316/2016 | A / H5N8
A/Chicken/Sweden/SVA161122KU0453/SZ0209317/2016 | A / H5N8
A/Chicken/Sweden/SVA161122KU0453/SZ0209318/2016 | A / H5N8
A/Chicken/Sweden/SVA161122KU0453/SZ0209321/2016 | A / H5N8
A/Common Goldeneye/Sweden/SVA161117KU0322/SZ0002165/2016 | A / H5N8
A/duck/Mongolia/179/2015 | A / H3N8
A/duck/Bangladesh/26992/2015(H7N9)
A/duck/Bangladesh/27042/2015(H7N9)
A/duck/Bangladesh/26980/2015(H7N9)
A/environment/Kamchatka/24/2016 | A / H13N8
A/mallard/Republic of Georgia/13/2011 | A / H6N2
A/duck/Hunan/S11893/2012 | A / H4N6
A/duck/Bangladesh/24694/2015(H7N1)
A/duck/Bangladesh/24705/2015(H7N1)
A/black-tailed godwit/Bangladesh/24734/2015(H7N5)
A/duck/Bangladesh/24035(H10N1)
A/duck/Bangladesh/24697/2015(H15N9)
A/duck/Bangladesh/24704/2015(H15N9)
A/duck/Bangladesh/26920/2015(H3N6)
A/duck/Bangladesh/26948/2015(H3N6)
A/duck/Bangladesh/26974/2015(H3N6)
A/duck/Mongolia/518/2015 | A / H10N3
A/duck/Mongolia/211/2015 | A / H3N8
A/duck/Mongolia/83/2015 | A / H3N8
A/duck/Mongolia/30/2015 | A / H3N8
A/duck/Mongolia/777/2015 | A / H3N8
A/duck/Mongolia/742/2015 | A / H10N7
A/duck/Mongolia/655/2015 | A / H2N3
A/teal/Chany/444/2009 | A / H8N8
A/greylag goose/Iceland/0976/2011 | A / H6N8
A/pink-footed goose/Iceland/0987/2011 | A / H6N8
A/greylag goose/Iceland/0961/2011 | A / H6N8
A/greylag goose/Iceland/0980/2011 | A / H6N8
A/greylag goose/Iceland/1474/2011 | A / H6N8
A/garganey/Altai/1213/2007 | A / H5N2
A/mallard/Republic of Georgia/1/2012 | A / H1N1
A/mallard/Republic of Georgia/14/2011 | A / H10N7
A/mallard/Sweden/52405/2006 | A
A/duck/Bangladesh/24692/2015(H7N1)
A/duck/Bangladesh/24706/2015(H7N1)
A/duck/Guangxi/1157/2006 | A / H6N2
A/duck/Guangxi/1248/2006 | A / H6N2
A/duck/Guangxi/1074/2006 | A / H6N2
A/wild duck/Korea/SH60/2004 | A / H1N1
A/wild duck/Korea/CSM38/2004 | A / H1N1
A/aquatic bird/Korea/w74/2005 | A / H5N2
A/duck/Jiang Xi/2374/2005 | A / H3N6
A/wild waterfowl/Dongting/C2383/2012 | A / H1N2
A/duck/Sichuan/S4202/2011 | A / H4N2
A/mallard/Czech Republic/13438-29K/2010 | A / H11N9
A/environment/Chongqing/02412/2012 | A / H5N1
A/tufted duck/Republic of Georgia/1/2012 | A / H2N3
A/northern shoveler/Georgia/1/2010 | A / H2N3
A/common coot/Republic of Georgia/1/2010 | A / H6N2
A/mallard/Republic of Georgia/1/2010 | A / H10N7
A/mallard/Republic of Georgia/2/2012 | A / H2N3
A/garganey/Republic of Georgia/1/2011 | A / H4N2
A/grey heron /Uvs-Nuur Lake/20/2016 | A / H5N8
A/common tern /Uvs-Nuur Lake/26/2016 | A / H5N8
A/wild duck/Tyva/35/2016 | A / H5N8
A/great crested grebe/Tyva/34/2016 | A / H5N8
A/black-headed gull/Tyva/41/2016 | A / H5N8
A/great crested grebe/Uvs-Nuur Lake/341/2016 | A / H5N8
A/duck/Mongolia/996/2015 | A / H3N8
A/duck/Mongolia/118/2015 | A / H4N6
A/muscovy duck/Vietnam/LBM529/2013 | A / H3N8
A/duck/Mongolia/769/2015 | A / H4N6
A/duck/Mongolia/20/2015 | A / H3N8
A/duck/Mongolia/101/2015 | A / H4N6
A/duck/Mongolia/127/2015 | A / H4N6
A/duck/Mongolia/66/2015 | A / H10N2
A/duck/Mongolia/326/2015 | A / H10N3
A/duck/Mongolia/520/2015 | A / H1N1
A/chicken/Korea/HN1/2016(H5N6) | A / H5N6
A/duck/Korea/ES2/2016(H5N6) | A / H5N6
A/mandarin duck/Korea/WB246/2016(H5N6) | A / H5N6
Group A
Group B
Clade 2.3.4.4 H5N8 Subtype
Genotype 2 H5N8 Subtype
Genotype 1 H5N8 Subtype
Eurasian lineage
0.01

D NP

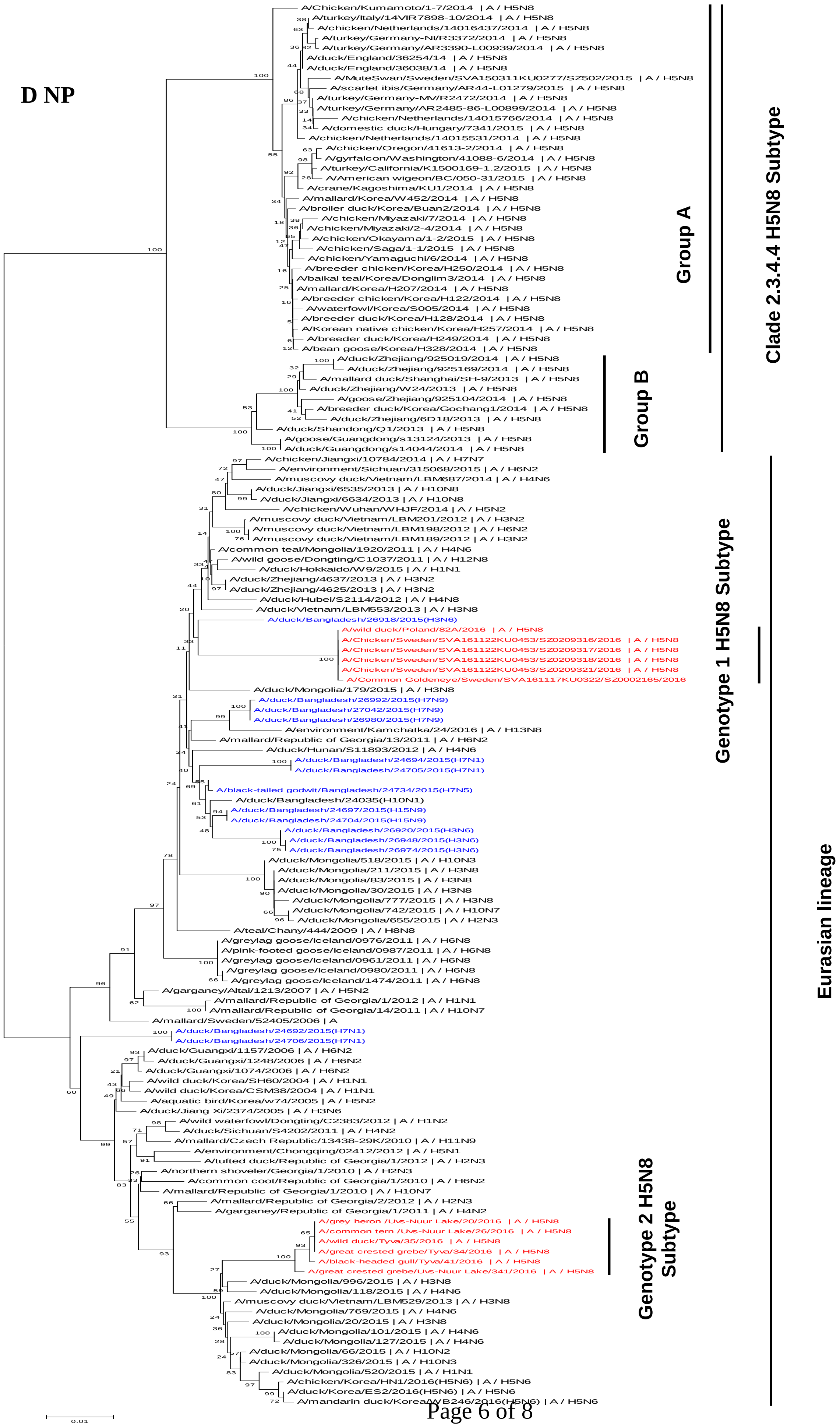
A/Chicken/Kumamoto/1-7/2014 | A / H5N8
A/turkey/Italy/14VIR7898-10/2014 | A / H5N8
A/chicken/Netherlands/14016437/2014 | A / H5N8
A/turkey/Germany-NI/R3372/2014 | A / H5N8
A/turkey/Germany/AR3390-L00939/2014 | A / H5N8
A/duck/England/36254/14 | A / H5N8
A/duck/England/36038/14 | A / H5N8
A/MuteSwan/Sweden/SVA150311KU0277/SZ502/2015 | A / H5N8
A/scarlet ibis/Germany/AR44-L01279/2015 | A / H5N8
A/turkey/Germany-MV/R2472/2014 | A / H5N8
A/turkey/Germany/AR2485-86-L00899/2014 | A / H5N8
A/chicken/Netherlands/14015766/2014 | A / H5N8
A/domestic duck/Hungary/7341/2015 | A / H5N8
A/chicken/Netherlands/14015531/2014 | A / H5N8
A/chicken/Oregon/41613-2/2014 | A / H5N8
A/gyrfalcon/Washington/41088-6/2014 | A / H5N8
A/turkey/California/K1500169-1.2/2015 | A / H5N8
A/American wigeon/BC/050-31/2015 | A / H5N8
A/crane/Kagoshima/KU1/2014 | A / H5N8
A/mallard/Korea/W452/2014 | A / H5N8
A/broiler duck/Korea/Buan2/2014 | A / H5N8
A/chicken/Miyazaki/7/2014 | A / H5N8
A/chicken/Miyazaki/2-4/2014 | A / H5N8
A/chicken/Okayama/1-2/2015 | A / H5N8
A/chicken/Saga/1-1/2015 | A / H5N8
A/chicken/Yamaguchi/6/2014 | A / H5N8
A/breeder chicken/Korea/H250/2014 | A / H5N8
A/baikal teal/Korea/Donglim3/2014 | A / H5N8
A/mallard/Korea/H207/2014 | A / H5N8
A/breeder chicken/Korea/H122/2014 | A / H5N8
A/waterfowl/Korea/S005/2014 | A / H5N8
A/breeder duck/Korea/H128/2014 | A / H5N8
A/Korean native chicken/Korea/H257/2014 | A / H5N8
A/breeder duck/Korea/H249/2014 | A / H5N8
A/bean goose/Korea/H328/2014 | A / H5N8
A/duck/Zhejiang/925019/2014 | A / H5N8
A/duck/Zhejiang/925169/2014 | A / H5N8
A/mallard duck/Shanghai/SH-9/2013 | A / H5N8
A/duck/Zhejiang/W24/2013 | A / H5N8
A/goose/Zhejiang/925104/2014 | A / H5N8
A/breeder duck/Korea/Gochang1/2014 | A / H5N8
A/duck/Zhejiang/6D18/2013 | A / H5N8
A/duck/Shandong/Q1/2013 | A / H5N8
A/goose/Guangdong/s13124/2013 | A / H5N8
A/duck/Guangdong/s14044/2014 | A / H5N8
A/chicken/Jiangxi/10784/2014 | A / H7N7
A/environment/Sichuan/315068/2015 | A / H6N2
A/muscovy duck/Vietnam/LBM687/2014 | A / H4N6
A/duck/Jiangxi/6535/2013 | A / H10N8
A/duck/Jiangxi/6634/2013 | A / H10N8
A/chicken/Wuhan/WHJF/2014 | A / H5N2
A/muscovy duck/Vietnam/LBM201/2012 | A / H3N2
A/muscovy duck/Vietnam/LBM198/2012 | A / H6N2
A/muscovy duck/Vietnam/LBM189/2012 | A / H3N2
A/common teal/Mongolia/1920/2011 | A / H4N6
A/wild goose/Dongting/C1037/2011 | A / H12N8
A/duck/Hokkaido/W9/2015 | A / H1N1
A/duck/Zhejiang/4637/2013 | A / H3N2
A/duck/Zhejiang/4625/2013 | A / H3N2
A/duck/Hubei/S2114/2012 | A / H4N8
A/duck/Vietnam/LBM553/2013 | A / H3N8
A/duck/Bangladesh/26918/2015(H3N6)
A/wild duck/Poland/82A/2016 | A / H5N8
A/Chicken/Sweden/SVA161122KU0453/SZ0209316/2016 | A / H5N8
A/Chicken/Sweden/SVA161122KU0453/SZ0209317/2016 | A / H5N8
A/Chicken/Sweden/SVA161122KU0453/SZ0209318/2016 | A / H5N8
A/Chicken/Sweden/SVA161122KU0453/SZ0209321/2016 | A / H5N8
A/Common Goldeneye/Sweden/SVA161117KU0322/SZ0002165/2016
A/duck/Mongolia/179/2015 | A / H3N8
A/duck/Bangladesh/26992/2015(H7N9)
A/duck/Bangladesh/27042/2015(H7N9)
A/duck/Bangladesh/26980/2015(H7N9)
A/environment/Kamchatka/24/2016 | A / H13N8
A/mallard/Republic of Georgia/13/2011 | A / H6N2
A/duck/Hunan/S11893/2012 | A / H4N6
A/duck/Bangladesh/24694/2015(H7N1)
A/duck/Bangladesh/24705/2015(H7N1)
A/black-tailed godwit/Bangladesh/24734/2015(H7N5)
A/duck/Bangladesh/24035(H10N1)
A/duck/Bangladesh/24697/2015(H15N9)
A/duck/Bangladesh/24704/2015(H15N9)
A/duck/Bangladesh/26920/2015(H3N6)
A/duck/Bangladesh/26948/2015(H3N6)
A/duck/Bangladesh/26974/2015(H3N6)
A/duck/Mongolia/518/2015 | A / H10N3
A/duck/Mongolia/211/2015 | A / H3N8
A/duck/Mongolia/83/2015 | A / H3N8
A/duck/Mongolia/30/2015 | A / H3N8
A/duck/Mongolia/777/2015 | A / H3N8
A/duck/Mongolia/742/2015 | A / H10N7
A/duck/Mongolia/655/2015 | A / H2N3
A/teal/Chany/444/2009 | A / H8N8
A/greylag goose/Iceland/0976/2011 | A / H6N8
A/pink-footed goose/Iceland/0987/2011 | A / H6N8
A/greylag goose/Iceland/0961/2011 | A / H6N8
A/greylag goose/Iceland/0980/2011 | A / H6N8
A/greylag goose/Iceland/1474/2011 | A / H6N8
A/garganey/Altai/1213/2007 | A / H5N2
A/mallard/Republic of Georgia/1/2012 | A / H1N1
A/mallard/Republic of Georgia/14/2011 | A / H10N7
A/mallard/Sweden/52405/2006 | A
A/duck/Bangladesh/24692/2015(H7N1)
A/duck/Bangladesh/24706/2015(H7N1)
A/duck/Guangxi/1157/2006 | A / H6N2
A/duck/Guangxi/1248/2006 | A / H6N2
A/duck/Guangxi/1074/2006 | A / H6N2
A/wild duck/Korea/SH60/2004 | A / H1N1
A/wild duck/Korea/CSM38/2004 | A / H1N1
A/aquatic bird/Korea/w74/2005 | A / H5N2
A/duck/Jiang Xi/2374/2005 | A / H3N6
A/wild waterfowl/Dongting/C2383/2012 | A / H1N2
A/duck/Sichuan/S4202/2011 | A / H4N2
A/mallard/Czech Republic/13438-29K/2010 | A / H11N9
A/environment/Chongqing/02412/2012 | A / H5N1
A/tufted duck/Republic of Georgia/1/2012 | A / H2N3
A/northern shoveler/Georgia/1/2010 | A / H2N3
A/common coot/Republic of Georgia/1/2010 | A / H6N2
A/mallard/Republic of Georgia/1/2010 | A / H10N7
A/mallard/Republic of Georgia/2/2012 | A / H2N3
A/garganey/Republic of Georgia/1/2011 | A / H4N2
A/grey heron /Uvs-Nuur Lake/20/2016 | A / H5N8
A/common tern /Uvs-Nuur Lake/26/2016 | A / H5N8
A/wild duck/Tyva/35/2016 | A / H5N8
A/great crested grebe/Tyva/34/2016 | A / H5N8
A/black-headed gull/Tyva/41/2016 | A / H5N8
A/great crested grebe/Uvs-Nuur Lake/341/2016 | A / H5N8
A/duck/Mongolia/996/2015 | A / H3N8
A/duck/Mongolia/118/2015 | A / H4N6
A/muscovy duck/Vietnam/LBM529/2013 | A / H3N8
A/duck/Mongolia/769/2015 | A / H4N6
A/duck/Mongolia/20/2015 | A / H3N8
A/duck/Mongolia/101/2015 | A / H4N6
A/duck/Mongolia/127/2015 | A / H4N6
A/duck/Mongolia/66/2015 | A / H10N2
A/duck/Mongolia/326/2015 | A / H10N3
A/duck/Mongolia/520/2015 | A / H1N1
A/chicken/Korea/HN1/2016(H5N6) | A / H5N6
A/duck/Korea/ES2/2016(H5N6) | A / H5N6
A/mandarin duck/Korea/WB246/2016(H5N6) | A / H5N6
0.01
Group A
Group B
Clade 2.3.4.4 H5N8 Subtype
Genotype 1 H5N8 Subtype
Genotype 2 H5N8 Subtype
Eurasian lineage

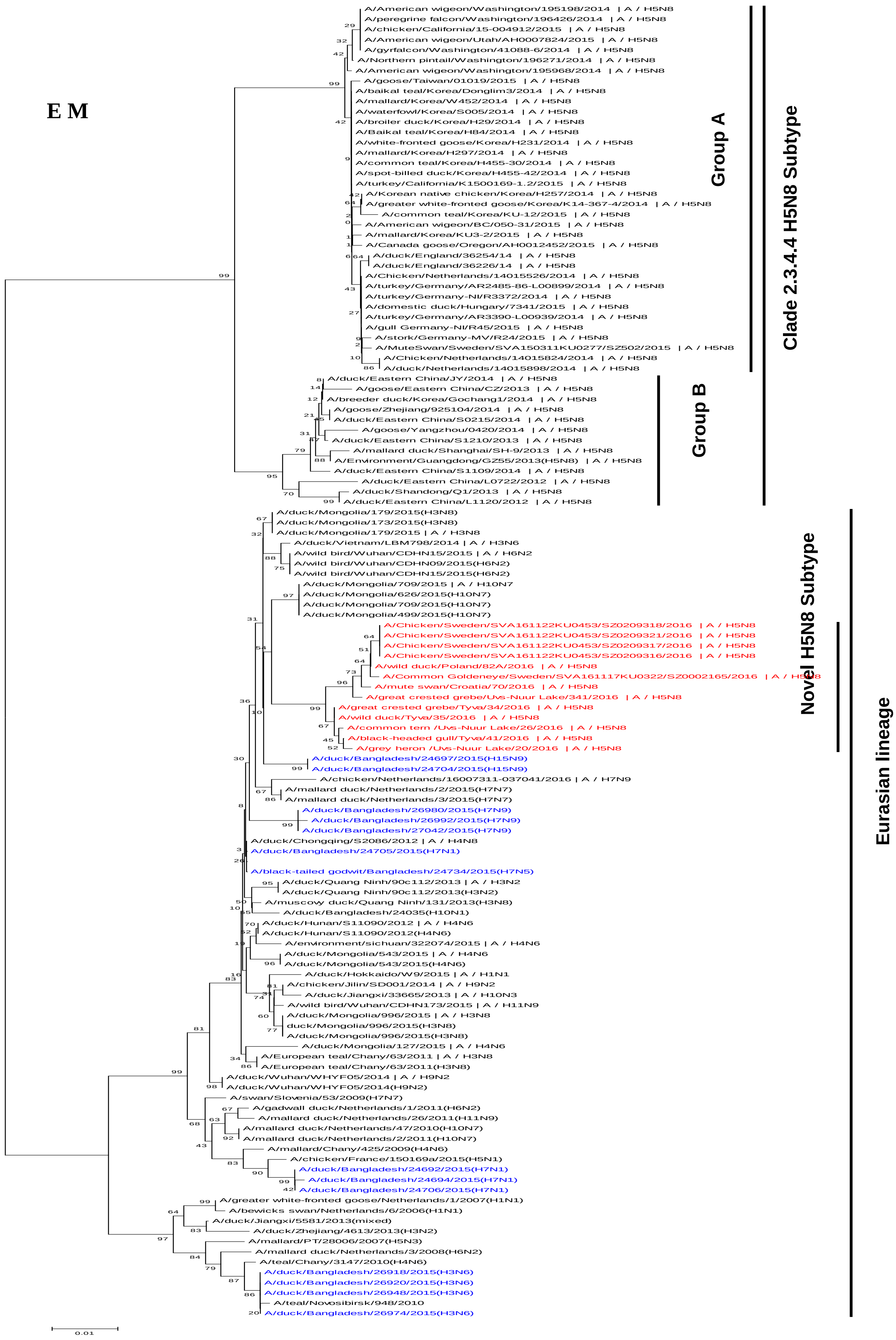

E M
Group A
Group B
Clade 2.3.4.4 H5N8 Subtype
Novel H5N8 Subtype
Eurasian lineage
0.01

F NS

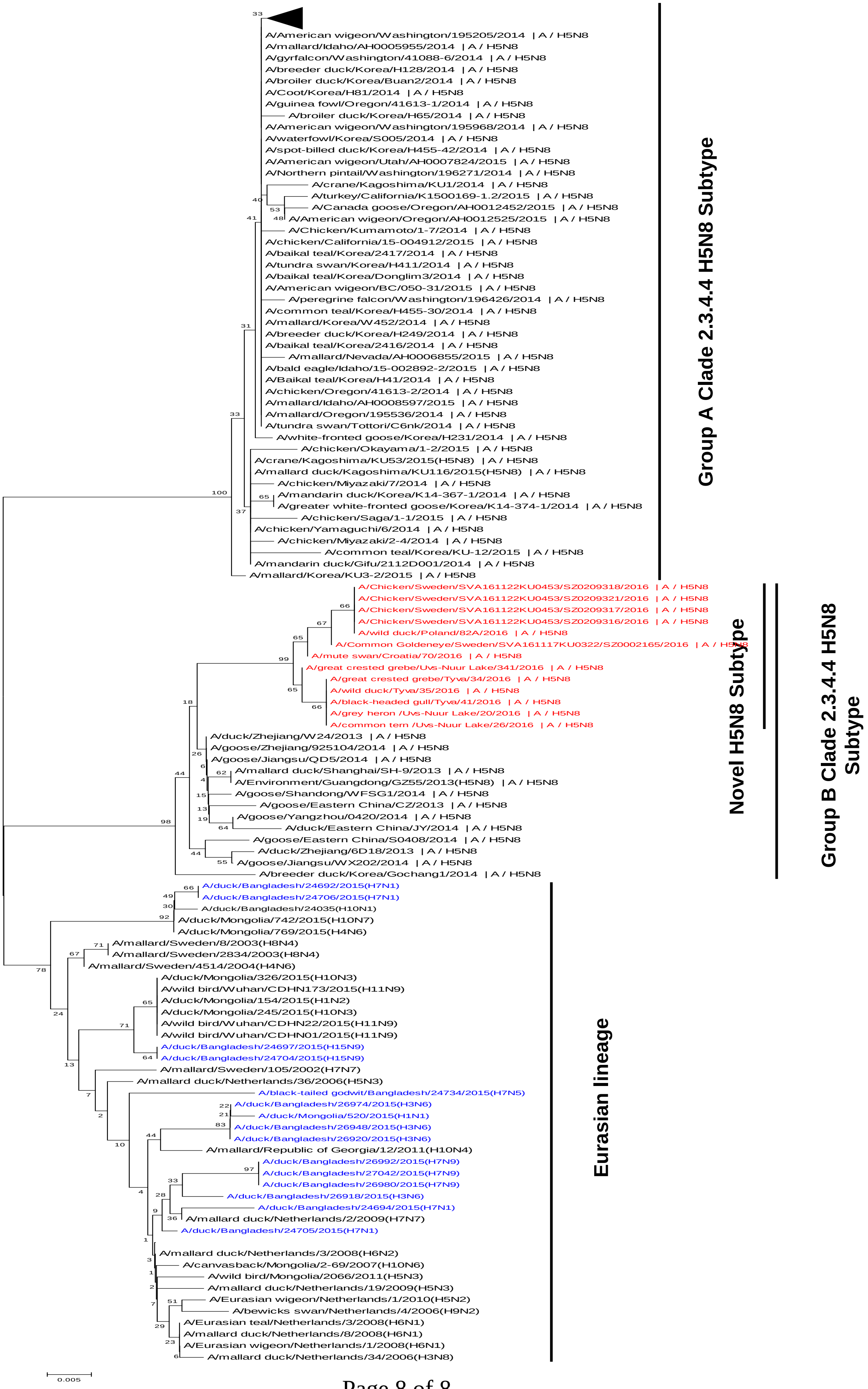
A/American wigeon/Washington/195205/2014 | A / H5N8
A/mallard/Idaho/AH0005955/2014 | A / H5N8
A/gyrfalcon/Washington/41088-6/2014 | A / H5N8
A/breeder duck/Korea/H128/2014 | A / H5N8
A/broiler duck/Korea/Buan2/2014 | A / H5N8
A/Coot/Korea/H81/2014 | A / H5N8
A/guinea fowl/Oregon/41613-1/2014 | A / H5N8
A/broiler duck/Korea/H65/2014 | A / H5N8
A/American wigeon/Washington/195968/2014 | A / H5N8
A/waterfowl/Korea/S005/2014 | A / H5N8
A/spot-billed duck/Korea/H455-42/2014 | A / H5N8
A/American wigeon/Utah/AH0007824/2015 | A / H5N8
A/Northern pintail/Washington/196271/2014 | A / H5N8
A/crane/Kagoshima/KU1/2014 | A / H5N8
A/turkey/California/K1500169-1.2/2015 | A / H5N8
A/Canada goose/Oregon/AH0012452/2015 | A / H5N8
A/American wigeon/Oregon/AH0012525/2015 | A / H5N8
A/Chicken/Kumamoto/1-7/2014 | A / H5N8
A/chicken/California/15-004912/2015 | A / H5N8
A/baikal teal/Korea/2417/2014 | A / H5N8
A/tundra swan/Korea/H411/2014 | A / H5N8
A/baikal teal/Korea/Donglim3/2014 | A / H5N8
A/American wigeon/BC/050-31/2015 | A / H5N8
A/peregrine falcon/Washington/196426/2014 | A / H5N8
A/common teal/Korea/H455-30/2014 | A / H5N8
A/mallard/Korea/W452/2014 | A / H5N8
A/breeder duck/Korea/H249/2014 | A / H5N8
A/baikal teal/Korea/2416/2014 | A / H5N8
A/mallard/Nevada/AH0006855/2015 | A / H5N8
A/bald eagle/Idaho/15-002892-2/2015 | A / H5N8
A/Baikal teal/Korea/H41/2014 | A / H5N8
A/chicken/Oregon/41613-2/2014 | A / H5N8
A/mallard/Idaho/AH0008597/2015 | A / H5N8
A/mallard/Oregon/195536/2014 | A / H5N8
A/tundra swan/Tottori/C6nk/2014 | A / H5N8
A/white-fronted goose/Korea/H231/2014 | A / H5N8
A/chicken/Okayama/1-2/2015 | A / H5N8
A/crane/Kagoshima/KU53/2015(H5N8) | A / H5N8
A/mallard duck/Kagoshima/KU116/2015(H5N8) | A / H5N8
A/chicken/Miyazaki/7/2014 | A / H5N8
A/mandarin duck/Korea/K14-367-1/2014 | A / H5N8
A/greater white-fronted goose/Korea/K14-374-1/2014 | A / H5N8
A/chicken/Saga/1-1/2015 | A / H5N8
A/chicken/Yamaguchi/6/2014 | A / H5N8
A/chicken/Miyazaki/2-4/2014 | A / H5N8
A/common teal/Korea/KU-12/2015 | A / H5N8
A/mandarin duck/Gifu/2112D001/2014 | A / H5N8
A/mallard/Korea/KU3-2/2015 | A / H5N8
Group A Clade 2.3.4.4 H5N8 Subtype
A/Chicken/Sweden/SVA161122KU0453/SZ0209318/2016 | A / H5N8
A/Chicken/Sweden/SVA161122KU0453/SZ0209321/2016 | A / H5N8
A/Chicken/Sweden/SVA161122KU0453/SZ0209317/2016 | A / H5N8
A/Chicken/Sweden/SVA161122KU0453/SZ0209316/2016 | A / H5N8
A/wild duck/Poland/82A/2016 | A / H5N8
A/Common Goldeneye/Sweden/SVA161117KU0322/SZ0002165/2016 | A / H5N8
A/mute swan/Croatia/70/2016 | A / H5N8
A/great crested grebe/Uvs-Nuur Lake/341/2016 | A / H5N8
A/great crested grebe/Tyva/34/2016 | A / H5N8
A/wild duck/Tyva/35/2016 | A / H5N8
A/black-headed gull/Tyva/41/2016 | A / H5N8
A/grey heron /Uvs-Nuur Lake/20/2016 | A / H5N8
A/common tern /Uvs-Nuur Lake/26/2016 | A / H5N8
A/duck/Zhejiang/W24/2013 | A / H5N8
A/goose/Zhejiang/925104/2014 | A / H5N8
A/goose/Jiangsu/QD5/2014 | A / H5N8
A/mallard duck/Shanghai/SH-9/2013 | A / H5N8
A/Environment/Guangdong/GZ55/2013(H5N8) | A / H5N8
A/goose/Shandong/WFSG1/2014 | A / H5N8
A/goose/Eastern China/CZ/2013 | A / H5N8
A/goose/Yangzhou/0420/2014 | A / H5N8
A/duck/Eastern China/JY/2014 | A / H5N8
A/goose/Eastern China/S0408/2014 | A / H5N8
A/duck/Zhejiang/6D18/2013 | A / H5N8
A/goose/Jiangsu/WX202/2014 | A / H5N8
A/breeder duck/Korea/Gochang1/2014 | A / H5N8
Novel H5N8 Subtype
Group B Clade 2.3.4.4 H5N8 Subtype
A/duck/Bangladesh/24692/2015(H7N1)
A/duck/Bangladesh/24706/2015(H7N1)
A/duck/Bangladesh/24035(H10N1)
A/duck/Mongolia/742/2015(H10N7)
A/duck/Mongolia/769/2015(H4N6)
A/mallard/Sweden/8/2003(H8N4)
A/mallard/Sweden/2834/2003(H8N4)
A/mallard/Sweden/4514/2004(H4N6)
A/duck/Mongolia/326/2015(H10N3)
A/wild bird/Wuhan/CDHN173/2015(H11N9)
A/duck/Mongolia/154/2015(H1N2)
A/duck/Mongolia/245/2015(H10N3)
A/wild bird/Wuhan/CDHN22/2015(H11N9)
A/wild bird/Wuhan/CDHN01/2015(H11N9)
A/duck/Bangladesh/24697/2015(H15N9)
A/duck/Bangladesh/24704/2015(H15N9)
A/mallard/Sweden/105/2002(H7N7)
A/mallard duck/Netherlands/36/2006(H5N3)
A/black-tailed godwit/Bangladesh/24734/2015(H7N5)
A/duck/Bangladesh/26974/2015(H3N6)
A/duck/Mongolia/520/2015(H1N1)
A/duck/Bangladesh/26948/2015(H3N6)
A/duck/Bangladesh/26920/2015(H3N6)
A/mallard/Republic of Georgia/12/2011(H10N4)
A/duck/Bangladesh/26992/2015(H7N9)
A/duck/Bangladesh/27042/2015(H7N9)
A/duck/Bangladesh/26980/2015(H7N9)
A/duck/Bangladesh/26918/2015(H3N6)
A/duck/Bangladesh/24694/2015(H7N1)
A/mallard duck/Netherlands/2/2009(H7N7)
A/duck/Bangladesh/24705/2015(H7N1)
A/mallard duck/Netherlands/3/2008(H6N2)
A/canvasback/Mongolia/2-69/2007(H10N6)
A/wild bird/Mongolia/2066/2011(H5N3)
A/mallard duck/Netherlands/19/2009(H5N3)
A/Eurasian wigeon/Netherlands/1/2010(H5N2)
A/bewicks swan/Netherlands/4/2006(H9N2)
A/Eurasian teal/Netherlands/3/2008(H6N1)
A/mallard duck/Netherlands/8/2008(H6N1)
A/Eurasian wigeon/Netherlands/1/2008(H6N1)
A/mallard duck/Netherlands/34/2006(H3N8)
Eurasian lineage
